# Supplementary material for: How ready is the health care system in Northeast India for surgical delivery? a mixed-methods study on surgical capacity and need
Source: PLoS One. 2024 Jun 26;19(6):e0287941. doi: 10.1371/journal.pone.0287941 (PMC11206862; doi:10.1371/journal.pone.0287941)
Supplement: S1 File — (DOCX) [file pone.0287941.s001.docx]

**Supplementary table: Profile of interviewed doctors**

| **Designation/Category** | **Gender, age** | **Category of Facility (Public/private, mission)** | **District** | **State** |
| --- | --- | --- | --- | --- |
| MP (Medical Provider) | Male, 30-35 | Private | Churachandpur | Manipur |
| MP (Medical Provider) | Male, 35-40 | Private | Churachandpur | Manipur |
| MP (Medical Provider) | Male, 35-40 | Public | Churachandpur | Manipur |
|  |  |  |  |  |
| MP (Medical Provider) | Male, 35-40 | Private | Bhaloukpong | Arunachal Pradesh |
| MP (Medical Provider) | Male, 35-40 | Private | Bongaigaon | Assam |
| MP (Medical Provider) | Male, 32-37 | Private | Chirang | Assam |
| MP (Medical Provider) | Male, 35-40 | Private | Dimapur | Nagaland |
| MP (Medical Provider) | Male, 40-45 | Private | Dimapur | Nagaland |
| MP (Medical Provider) | Male, 37 | Private | Dimapur | Nagaland |
