# Supplementary material for: How ready is the health care system in Northeast India for surgical delivery? a mixed-methods study on surgical capacity and need
Source: PLoS One. 2024 Jun 26;19(6):e0287941. doi: 10.1371/journal.pone.0287941 (PMC11206862; doi:10.1371/journal.pone.0287941)
Supplement: Attachment — Submitted filename: Response to Reviewers.docx [file pone.0287941.s003.docx]

To,

The Editor,

PLOS ONE

27 September 2023

**Subject: Revisions to (PONE-D-23-17522), ‘How ready is the health care system in North East India for surgical delivery? A mixed methods study on surgical capacity and need’.**

Dear Editor and Reviewers,

Many thanks for your careful consideration of our paper (PONE-D-23-17522), ‘How ready is the health care system in North East India for surgical delivery? A mixed methods study on surgical capacity and need.’ We are very grateful for the editor’s and reviewers’ very valuable comments to strengthen the manuscript.

We have appended responses to the comments raised below. As requested we have attached

1. A rebuttal letter that responds to each point raised by the academic editor and reviewer(s). We have uploaded this letter as a separate file labelled 'Response to Reviewers'.
2. A marked-up copy of the manuscript that highlights changes made to the original version. We have uploaded this as a separate file labelled 'Revised Manuscript with Track Changes'.
3. An unmarked version of your revised paper without tracked changes. You should upload this as a separate file labelled 'Manuscript (clean)'.

Kindly let us know if anything further is needed.

Best regards,

(On behalf of all authors)

Amrit Virk

Journal requirements:

When submitting your revision, we need you to address these additional requirements.

1. Please ensure that your manuscript meets PLOS ONE's style requirements, including those for file naming. The PLOS ONE style templates can be found at

https://journals.plos.org/plosone/s/file?id=wjVg/PLOSOne_formatting_sample_main_body.pdf and

<https://journals.plos.org/plosone/s/file?id=ba62/PLOSOne_formatting_sample_title_authors_affiliations.pdf>

Response: noted and to the best of our knowledge, PLOS ONE style requirements adhered to with respect for example, double spacing, inserting line numbers.

2. Please include a complete copy of PLOS’ questionnaire on inclusivity in global research in your revised manuscript. Our policy for research in this area aims to improve transparency in the reporting of research performed outside of researchers’ own country or community. The policy applies to researchers who have travelled to a different country to conduct research, research with Indigenous populations or their lands, and research on cultural artefacts. The questionnaire can also be requested at the journal’s discretion for any other submissions, even if these conditions are not met. Please find more information on the policy and a link to download a blank copy of the questionnaire here: https://journals.plos.org/plosone/s/best-practices-in-research-reporting. Please upload a completed version of your questionnaire as Supporting Information when you resubmit your manuscript.

Response: Noted, thank you. We have uploaded the questionnaire as Supporting Information.

3. We note that you have stated that you will provide repository information for your data at acceptance. Should your manuscript be accepted for publication, we will hold it until you provide the relevant accession numbers or DOIs necessary to access your data. If you wish to make changes to your Data Availability statement, please describe these changes in your cover letter and we will update your Data Availability statement to reflect the information you provide.

Response: We can upload the data to the Research Data Leeds Repository and make a DOI or URL available.

4. Your ethics statement should only appear in the Methods section of your manuscript. If your ethics statement is written in any section besides the Methods, please move it to the Methods section and delete it from any other section. Please ensure that your ethics statement is included in your manuscript, as the ethics statement entered into the online submission form will not be published alongside your manuscript.

Response: Noted, and ethics statement moved to appear in the methods section only.

5. Please include your tables as part of your main manuscript and remove the individual files. Please note that supplementary tables (should remain/ be uploaded) as separate "supporting information" files

Response: Included tables as part main manuscript and removed the individual files.

**Additional Editor Comments:**

The manuscript has good information on health system capacity in resource constraint settings in Northeast India; however, on other side it is a classic case of “Helicopter research” when researchers from higher-income or more privileged settings carry out research in resource-poor settings with limited to no involvement of local researchers. There is no Indian Co-author in the Author list.

The world is talking about “Equitable Research Partnerships” and new frameworks being developed to promote greater equity in global collaborations.

The manuscript suggests at many places about the work done by local team “The UK based team worked in close collaboration with regional institutions, including a clinician-led, humanitarian organization providing medical care and surgical training in the region and a team of social scientists from the region.” Despite this, local researchers are not part of the authorship.

Response: We fully appreciate and take on board the fact that the authors list can represent those in roles not directly concerned with manuscript preparation and the importance of including local authors. We would like to mention that the lead author (AV) is Indian and has lived and worked in the country for over 20 years, as has MH who led the work on the ground as founder and Director of the local institute in Kohima. However, in cognisance of the importance of naming data collectors in manuscripts, and mindful of ICMJE criteria for authorship, we have additionally included the two local researchers (LA and CC) who led the two arms of the study, and made inputs into the analysis. In compliance with ICJME guidance local researchers (MH, LA, CC) have also read and approved the final manuscript.

The IRB approval is matter of great concern as pointed by few reviewers because the so-called agency is located in New Delhi and is providing Approval for the study in different state. The approval letter says that it is Institutional Review Board but no Institutional attachment. The Indian Council of Medical Research (ICMR), New Delhi, the apex body in India for the formulation, coordination and promotion of biomedical research as per its guidelines provides unique registration number for the IRB and also the process for International Collaborations through HMSC, Health Ministry Screening committee as the local data has moved from the country to another country. Therefore, the authors must provide the proper approval documents; in the absence of which the manuscript cannot be considered further.

Response: Thank you for your response and for highlighting this. We appreciate your concerns and are happy to provide further information on this.

At the time of this study, there were no identifiable Institutional Review Boards operating in Arunachal Pradesh, Nagaland, or Manipur states; with IRBs in Assam focusing primarily on bio-medical research, with remits limited to Assam state. Given the anthropological aspects of the research, as well as its regional focus, advice was sought from the Tomo Riba Institute of Health and Medical Sciences (TRIHMS) in Arunachal Pradesh, as well as the Nagaland Health Project (NHP), in the selection of an IRB, and Sigma IRB came highly recommended. Sigma IRB while located in New Delhi, covers the entire country for IRB-related work. Sigma is registered with the NIH in the USA: please see: [Office for Human Research Protections Database (nih.gov)](https://ohrp.cit.nih.gov/search/search.aspx) (please click to access hyperlink), and focuses on social and behavioural studies. When Sigma IRB was started, ICMR registration was not yet open.

Compliance with European General Data Protection Regulation (GDPR) regulations was ensured. Data was thoroughly anonymised, analysed and encrypted before transmission overseas.

# **Reviewers' comments:**

# **Reviewer's Responses to Questions**

# **Comments to the Author**

1. Is the manuscript technically sound, and do the data support the conclusions?

The manuscript must describe a technically sound piece of scientific research with data that supports the conclusions. Experiments must have been conducted rigorously, with appropriate controls, replication, and sample sizes. The conclusions must be drawn appropriately based on the data presented.

Reviewer #1: Yes

Reviewer #2: Partly

Reviewer #3: Partly

Reviewer #4: Partly

Reviewer #5: Yes

Reviewer #6: Yes

Reviewer #7: No

Reviewer #8: Yes

2. Has the statistical analysis been performed appropriately and rigorously?

Reviewer #1: Yes

Reviewer #2: No

Reviewer #3: N/A

Reviewer #4: I Don't Know

Reviewer #5: N/A

Reviewer #6: Yes

Reviewer #7: N/A

Reviewer #8: I Don't Know

3. Have the authors made all data underlying the findings in their manuscript fully available?

The PLOS Data policy requires authors to make all data underlying the findings described in their manuscript fully available without restriction, with rare exception (please refer to the Data Availability Statement in the manuscript PDF file). The data should be provided as part of the manuscript or its supporting information, or deposited to a public repository. For example, in addition to summary statistics, the data points behind means, medians and variance measures should be available. If there are restrictions on publicly sharing data—e.g. participant privacy or use of data from a third party—those must be specified.

Reviewer #1: Yes

Reviewer #2: No

Reviewer #3: Yes

Reviewer #4: No

Reviewer #5: No

Reviewer #6: Yes

Reviewer #7: No

Reviewer #8: Yes

4. Is the manuscript presented in an intelligible fashion and written in standard English?

PLOS ONE does not copyedit accepted manuscripts, so the language in submitted articles must be clear, correct, and unambiguous. Any typographical or grammatical errors should be corrected at revision, so please note any specific errors here.

Reviewer #1: Yes

Reviewer #2: Yes

Reviewer #3: Yes

Reviewer #4: Yes

Reviewer #5: Yes

Reviewer #6: Yes

Reviewer #7: Yes

Reviewer #8: Yes

**5. Review Comments to the Author**

Please use the space provided to explain your answers to the questions above. You may also include additional comments for the author, including concerns about dual publication, research ethics, or publication ethics. (Please upload your review as an attachment if it exceeds 20,000 characters)

**Reviewer #1: I enjoyed reviewing this paper, because:**

1. The research question is well defined.

2. The methodology is appropriate.

3. Statistical analyses in result section are appropriate and figures and tables highlight the trends well.

4. In discussion authors have done well to discuss all relevant points and the limitations of the study are acknowledged.

My two major concerns are:

1. There is very little new in this manuscript; the deficiencies in rural remote areas are well known, as is the solution. However, lot of hard work has been put in this survey; hence it gets the nod from me.

2. A bigger concern is with the ‘Helicopter’ style of research in which there are no local authors, although all the data is ‘local’.

The call on the ethical suitability of such a paper being published in the journal has to be taken by the editorial team.

Response: We fully appreciate and take on board this comment and the importance of including local authors. We would like to mention that the lead author (AV) is Indian and has lived and worked in the country for over 20 years, as has MH who led the work on the ground as founder and Director of the local institute in Kohima. However, in cognisance of the importance of naming data collectors in manuscripts, we have additionally included the two local researchers (LA, CC) who led the two arms of the study and made inputs into the analysis. We would also like to mention that the local team were initially included as authors and when consulted, requested that they be listed in the acknowledgments section instead given their limited input into the writing of the text. The research team engages regularly with large-scale qualitative and quantitative health projects, often in collaboration with the Government of Nagaland’s Department of Health and Family Welfare, and co-authorship of reports is neither common nor expected. However, having considered your comments, we take on the board the fact that the authors list can represent those in roles not directly concerned with manuscript preparation. In compliance with ICJME guidance local researchers (MH, LA, CC) have also read and approved the final manuscript.

**Reviewer #2:**

1. The selected topic is relevant to policymakers and there is paucity of literature on it - therefore the study is justified.

Response: n/a

2. Considering a global audience for this topic, there should be a map as well as introduction to northeast India with a short description of its uniqueness (geography, ethnicity, history, factors such as insurgency etc.)

Response: We have added additional introductory information on the northeast India as advised (Pg 2) and inserted a Map 1, on page 4.

3. There are many factors that affect health-seeking behaviour, especially in remote rural locations in India. Risk taking behavior by hospitals/doctors (such as performance of anaesthesia by surgeons or referral to higher centers for trivial reasons) have been mentioned but these should be explored in detail in the design of the study. This is an article analysing some of these factors from northeast India: https://casereports.bmj.com/content/15/7/e248221 - this is a case report that analyses global health factors, there should be other studies as well.

Response: Thank you. We have now added a few sentences describing this and referencing to the study above. (Pg. 18 and Pg 22)

4. The sampled data is too small and not representative of the region. Data has been collected from only 4 districts/states, 8 doctors and 11 patients. There are several large hospitals both in the government and private sector which manage large volumes of patients. These should have been included.

Response: We appreciate that the sample is not large. However, for the survey, it includes all facilities that in those districts that were willing to participate. The purpose of the qualitative interviews was to look at more depth into some of the issues emerging from the survey rather than to develop generalizable data. Moreover, as described on page 2 of the manuscript, the study’s aim was not to generalise but instead provide evidence on the current state of surgical care in certain remote locations within these four states in order to develop a preliminary account and baseline for understanding the readiness and potential of facilities to deliver surgical care.

5. With a high 'out of pocket' expenditure in healthcare, Indian costs should be analysed from the patient's point of view. It will be good to visit large, medium and small government and private hospitals to make a detailed analysis of how much the patient has paid towards service - those admitted to government hospitals also spend significant amounts to purchase drugs/supplies or non-available diagnostics. They also face significant non-hospital expenditure such as travel, loss of wages, food etc.

Response: Thank you. This is a great suggestion, and we will consider this an area for future work.

6. Indian states have adopted their versions of the Clinical Establishment Act whienumerates a number of conditions that must be met for registration. Laws applicable to blood banking and ultrasound are unique to India and a major barrier in northeast India due to the paucity of pathologists and radiologists. These are an important barrier to service provision (especially in remote rural areas of the region). Besides electricity, water and oxygen, internet access could be considered as one of the critical requirements. It enables high quality second opinions and the ability to log in to government and private portals for the many health care initiatives that are being rolled out by the Government of India. Besides technical and cost issues, internet access in many parts of northeast India are cut by the government for prolonged periods of time to tackle insurgency and other security issues. Distance to nearest center offering the service for which the patient has been transferred would be an important factor to measure.

Response: thank you. On page 18, we have mentioned the value of internet connectivity as an enabler to obtain high quality second opinions and to access health care initiatives being rolled out by the Government of India.

7. Surgical workload should mention how many surgeries among how many surgeons over what time period. Workloads ranging from 25 to 3000 surgeries appears to refer to institutional numbers per year.

Response: Table 4 has been revised to include numbers of surgeons and workloads per surgeon and reference added in the text.

8. Considering a global readership, costs should be converted from INR to USD.

Response: We have revised table 6 with dollar values (Dec 2019 exchange rate). We used the rate of 71.5 INR to a USD.

9. A very similar study (Ensor T. et.al.) with some of the same authors explores obstetric and gynaecologic surgeries in the same region. It is a much more comprehensive and better designed study. This study could borrow design and analysis features from the earlier study but include other general surgeries or omit the obstetric and gynaecological surgeries to derive greater value.

Response: The earlier study focused on the demand for surgery using household data from several secondary sources. It had general workloads for facilities but no further detail. It did not make use of qualitative analysis to explore the reasons for variation in functionality and use of services. The current study attempted to collect data directly from facilities. The sample for the current study is much smaller since data collection across facilities in the region is expensive and time consuming.

10. India offers a comprehensive health insurance scheme. The RSBY scheme has been mentioned in the above study but this has been replaced by the Ayushman Bharat scheme which is modified by different states. Access to this scheme with its barriers and enablers, reimbursement time, opt-in/opt-out rates etc. would also affect surgical service accessibility.

Response: noted, as a comment on the Ensor T. et.al. paper mentioned in comment 9 above.

11. Fracture repair and laparotomy are very broad descriptions. It could be better to be more specific - appendectomy, laparoscopic cholecystectomy, open reduction and internal fixation of a trochanteric fracture, open mesh hernioplasty etc. would be good examples.

Response: Table 3 (pages 9-10) does have some detail, where laparotomy is separated from appendectomy, cholecystectomy for example. Here laparotomy is used as a broad descriptive for any procedure where the abdomen is opened up for investigation.

12. The list of references has several duplications (same article mentioned twice).

Response: thank you, we have deleted duplications in the reference list.

**Reviewer #3:**

This study entitled ‘How ready is the health care system in North East India for surgical delivery? A mixed methods study on surgical capacity and need’ addresses the scarcity and unequal access to surgical services in Northeast India, an area with a high surgical need but sub-par delivery rates. The study uses mixed methods, including facility-based census and interviews with surgeons and patients, the research provides valuable sub-regional data on surgical provisioning and need. The authors have shown commendable efforts in organizing the manuscript in a comprehensible manner. Still, certain suggestions require attention and should be rectified by the authors.

1. Page No. 3, What does author means about (Box 1), is it a typo? As the Box1 is cited in the manuscript but mentioned in the manuscript itself? The author should thoroughly review and ensure consistency throughout the manuscript.

Response: Thank you for pointing this out. We have amended this to ‘Panel 2’.

2. Page No. 5, The authors have discussed the interviews conducted in Nagaland, Assam, and Manipur, but they have not mentioned anything about the interviews in Arunachal Pradesh, even though this state was included as one of the four selected states for the current study. Additionally, authors should justify about the exclusion of interview in Arunachal Pradesh.

Response: Please refer to page 11 which cites from the interview conducted with a surgeon from Arunachal Pradesh.

3. Authors have cited Panel 1 at two distinct places. Cite at the relevant place only.

Response: Panel 1 is cited once on page 4.

4. The authors are advised to verify the citation given on Page No. 5 under the section 'Site selection' for SRQR, as it is challenging for the reviewer to evaluate the specific details of the methods used in this study.

Response: added to reference list.

5. Is the data provided in Table 3 applicable to all four states? If so, it would be better to present the data separately for each state. By doing this, it will enable a state-wise comparison and facilitate better interpretation of the healthcare facility across the states.

Response: The sample was calculated for the NE states as a whole and so is not statistically representative at the state level. We feel, therefore, that reporting state level figures would be misleading and inaccurate.

6. The total referrals calculation in Table 5 seems to be incorrect. The authors are strongly encouraged to thoroughly review all the calculations for accuracy. Furthermore, it is suggested that the authors should rectify the percentage calculation in the Referrals/admission column, as it may be confusing. For example, the reported 5.5% for the Rural population in the Referrals/admission column requires clarification.

Response: Thanks for noting this. The percentages were worked out by averaging the percentages across the facilities rather than obtaining the percentage for total referrals and surgeries for the sample a whole. So the there is no inbuilt weighting for size of facility. We accept this is misleading and have now redrafted the table computing percentages for the totals as a whole. The references have been changed in the narrative.

7. Also, it would be better to mention about the number of the patient out of total Admissions that were excluded from the referrals and that were underwent surgeries.

Response: We would request further clarity on this comment please.

**Reviewer #4:**

Evaluation of data collected by 7 member team of local researchers in India has been done by authors based in the United Kingdom without anyone of them having any personal interaction with the study population. There was no uniformity in the doctors and patients interviewed, majority were from private sector.The sector of population , facilities and medical personel covered though mentioned as being uniform is not so. More so because data collected has been transmitted by a intermediary.

Response: MH is the founder and director of the local organisation, someone who has spent over 20 years living and working in the region, and oversaw the entire process from start to completion. Moreover, AV, TE, and MH undertook extensive and extended training (both in person as well as through regular fortnightly calls for the duration of the study) with the seven member local team which had limited to no prior experience conducting surveys and using qualitative methods. This involved AV and TE travelling to Nagaland and Arunachal Pradesh to develop local networks with potential respondents as well as with the local research team. Moreover, AV travelled to Nagaland and worked with MH, LA, CC and the local team to refine the topic guides and carry out the pilot interviews. The premise of the study was to ensure that local stakeholders led the work on the ground, and to build research capacity to undertake similar global health work independently. We have added this clarification into the manuscript on page 4, under ‘Study design’.

The study aimed for maximum variation to ensure a broad range of voices were represented. Anyone familiar with this part of the country will be aware of the difficult geographical terrain, the political sensitivity of the region, and the ethno-linguistic diversity here, which involved extensive negotiation of the part of the local team with facilities to access interviewees. Certain public and military facilities were not forthcoming given the political sensitivity of the region.

**Reviewer #5:**

Summary: The manuscript presents a mixed methods study investigating various aspects of surgical care service delivery & impact in Northeast India. I congratulate the authors on conducting a valuable study in a geographic region that requires more attention in global surgery. The study brings out rich data. I don't have any major concerns/issues. However, I believe that the manuscript could benefit from the following suggestions. All the best to the authors!

Comments:

1. Citations in multiple places seem misplaced. E.g., in the Introduction section, “Yet, a staggering 5 billion people – approximately 5 of every 7 people globally – lack access to safe, timely, and affordable surgical care (Jamison et al 2013; Debas et al 2015)” should cite Alkire et al. 2015 (https://pubmed.ncbi.nlm.nih.gov/25926087/) that gave the 5 billion number for access. Similarly, the statement - “The figures reveal vast geographical disparities, 295 in LMICs against 23,000 surgeries per 100,000 population in high-income countries, driven by shortages of medical staff, poor access to healthcare services, and weak record keeping (Bhandarkar et al 2021)” also does not seem to have an appropriate citation. Bhandarkar et al is about one tertiary hospital in India and could not have given numbers on average surgical volumes in HICs vs. LMICs. Where are the 295 and 23000 numbers from? I would suggest that authors cite the source.

Response: We have inserted the Alkire et al reference on page 2. The source for the 295 vs. 23000 is indeed Bhandarkar et al. We have added a reference to the primary document, Meara JG, Leather AJM, Hagander L, Alkire BC, Alonso N, Ameh EA, et al. Global Surgery 2030: evidence and solutions for achieving health, welfare, and economic development. The Lancet. 2015;386(9993):569–624.

2. Discussion and Conclusion in the abstract should start with an uppercase letter - “Skewed workloads …”

Response: We have addressed this in the abstract.

3. In the Introduction, “This region is also distinct in Eurasia, characterized by ethnolinguistic diversity, indicating that many vernacular health systems interact (Post et al 2022)” - I am unsure if the term “Eurasia” is appropriate here.

Response: thanks, we were citing from the source above, but have changed ‘Eurasia’ to ‘Asia’.

4. In Methods, the authors point to Box 1 which has the survey tool. But I could not find the box in the manuscript.

Response: Panel 2 on the facility functionality criteria added on page 7.

5. Similar to the profiles of patients interviewed in Table 2, information on doctors would be important - degree qualification (MBBS vs. MS/MD), subspeciality, years of practicing experience, gender, etc.

Response: We have provided a supplementary table, ‘Profile of interviewed doctors’.

6. Under the Methods - Facility Survey section, the authors mention - “Data entry was double-entry and undertaken by the local research team.” What do authors mean by double entry here? A clarification would be helpful for the readers.

Response: We have added a clarification on page 7 to say that data were entered by two people separately and any differences resolved.

7. Table 3 has percentages added across rows. However, it might be also interesting to have the percentages across columns for depicting public-private comparisons. Maybe another table or a bar chart could do that.

Response: We have added a column showing the % of each surgery carried out in private facilities and referenced this in the narrative.

8. The current Table 3 can be cleaned up to have just three columns for the variables instead of six. Add the percentages in parentheses in the same cell. E.g., 2289 (30%).

Response: We have cleaned up table 3 in the way the reviewer suggests. Thank you.

9. I would recommend that authors use "public" & "private" consistently throughout the manuscript text, tables, figures, and panels to improve readability.

Response: we have changed ‘government’ to ‘public’ in the manuscript.

10. Figure 2 should mention a correlation value to quantify the association.

Response: We have added the R square in brackets.

11. Additionally, the caption needs correction - “Figure 2: Number of different surgical procedures and facility functionality”

Response: We have corrected the caption for Figure 2 to ‘Number of different surgical procedures and facility functionality”

12. Table 4 should specify what values are being noted for Surgeons, Anesthetists, and other variables. Are those mean values or median? Otherwise, 3.8 surgeons - a fractional number - is confusing.

Response: They are mean values. We have added this to the table title.

13. Table 4 can be made concise by having min-max values in the same cell as that of the summary value. E.g., 3.8 (0.5, 9.0). That will reduce the number of rows by a third & make the table more readable.

Response: Thank you for this suggestion. We have done as suggested.

14. Table 6 can be cleaned up in the same way as Table 4. E.g., Average charges (min, max) = 27,700 (1400, 40,000).

Response: Re formatted as suggested. Thank you.

15. Also, ideally, it would be better to transpose the table. I.e., switch columns and rows after reducing the cells in an above-mentioned manner. That way, it'll have 3 rows as procedures & 2 columns as average (min, max) and %gdp per capita values.

Response: Since another reviewer has asked for dollars to be incorporated into the table we feel it is better not to transpose the table.

16. Is the GDP here Indian GDP or the average GDP of NE states? Please specify.

Response: GDP per capita is for North-East States. This is now clarified.

17. Figure 3 requires an axis label for the Y axis.

Response: This has been added

18. Also, since these are % values, they can range only from 0 to 100%. Please limit the Y-axis accordingly.

Response: Limited to 100% as suggested.

19. In the first paragraph of the Discussion, the authors mention - "... and the readiness for adoption of new surgical innovation.". However, the aims and study data do not support that. Readiness would depend on some demonstration of clinicians' acceptability toward new technology. I would recommend rephrasing it.

Response: Thank you, on page 17, we have deleted text saying "... and the readiness for adoption of new surgical innovation."

20. In the second paragraph of the Discussion, the authors remark - "Abdominal conditions constituted a large portion of the overall…". However, it's not clear in the manuscript, especially in the Methods, what all conditions were included in the facility surveys & the classification was conducted. The Introduction also primarily focuses on Bellwether procedures that do not include abdominal conditions.

Response: Based on table 3 abdominal conditions make up 37% and obstetric/gynaecological conditions make up 50% of the surgery. We have modified the first sentence of the second paragraph to make this clear.

21. In the third paragraph of the Discussion, the authors write - "... interviewees generally reporting excessive workloads although the facility survey also revealed smaller surgical volumes at certain facilities." This is a truly interesting finding that points to some disconnect in the data on workload & perception of the workload. Could the authors please add a few competing hypotheses/rationale for why this might be happening?

Response: Thank you. We have added a statement on page 18.

22. The fourth paragraph of the Discussion - on task shifting - is quite well described. A couple of pointers that the authors can choose to include. First, is there a way to measure task-shifting and -sharing? Can authors perhaps add a metric in their indicators to capture that? If so, that would be a valuable addition that will enhance the study. Second, can the authors discuss the regulatory context? Are nurses allowed to help in or provide anesthesia? Do the findings of this study support changes in regulations that would permit nurses and non-specialist doctors to take up better roles in surgical care? Comments on such areas would make the discussion better.

Response: Thank you. We have added a further paragraph on page 18.

23. I would recommend that authors add a paragraph in the Discussion that clearly highlights/summarizes the rural-urban & public-private differences for various parameters. The sixth paragraph does this partially but only in the context of referrals. The comparisons form an important takeaway of the valuable data cited in the manuscript that should be reiterated for the readers.

Response: We have added a paragraph in the discussion on this. (pg.19)

24. In Study Limitations, authors call upon their findings as "broadly representative". I would recommend using a more appropriate alternative as collecting representative data is not typically supported by the design of a mixed methods study. Authors should also consider changing any other such mentions of representativeness throughout the manuscript.

Response: We have replaced mention of ‘representative’ to ‘indicative’ (on page 19) and ‘describing’ rather than ‘representative of’ on page 3.

25. The last six references seem to have been numbered (unlike others) in the bibliography which may not be the correct numbers. I would recommend proofreading the manuscript once before resubmission.

Response: We have deleted these. Thank you.

Points of appreciation:

26. The manuscript flow is quite good in most places.
27. The figures are easy to comprehend and present the main takeaways clearly.
28. The facility functionality indicators are a great addition and I hope other researchers apply them in their work going ahead.

**Reviewer #6:**

Thank you for giving me the opportunity to review this excellent manuscript. The study design and approach is sound, giving a reliable view of the situation in the area under study. I have the following minor comments that may help improve the manuscript:

1. From the introduction: "Three procedures, caesarean section, fracture repair, and laparotomy together known as the Bellwether Procedures can treat a vast majority of surgical problems; ensuring they are available 24/7 at all first level hospitals a key strategy to expand surgical access (O’Neill 2016)". I am sure the authors are fully aware that more than directly treating surgical problems directly, the capacity to safely conduct these procedures translates into the ability of the surgical system to take care of the majority of first level procedures (for example, if a laparotomy can be performed, then abscess drainage, circumcision, chest tube placement, etc can also be conducted safely). The Bellwethers thus act as “proxy” or indicator surgical procedures. This is an important nuance that needs to be laid out clearly for the reader who may be new to this space.

Response: Thank you for making this important point. We have added in information (pg. 2) to make this nuanced point.

2. Is there any way to better chart the sub-regional area studies? Geographic area? Total population of the states under review as a percentage of the overall population of the country? Some general information that would better define the scope of the study, and in turn help the reader to understand its contribution to the overall health scenario of this very large country.

Response: We have added a sentences on page 2 to indicate the NE states’ geographic area and total population as a percentage of the overall population of the country.

3. Patients from private facilities; 9 out of 11 were women. Were any steps taken at the study design level to ensure a better gender representation? That may have added valuable insights.

Response: This is a great point, thank you. The team, while appreciative of the gender imbalance, were not seeking to overcome this. However, women significantly outnumber men in health facilities in indigenous/rural areas, largely related to women's health needs already mentioned, but it is also for cultural reasons. Indigenous men rarely seek medical treatment, unless in absolute emergency situations, such as accidents, or as a last resort in cases of disease, etc. More research to understand this dynamic may be required, specifically of a medical anthropological nature.

4. Consider using the term anesthesiologists instead of anesthetists or anesthetics (not sure what the latter term means).

Response: Change made throughout manuscript. (pg 7-9)

5. Page 12: "Although these results will need to be read with caution as there were significantly fewer (n=5) public facilities among the universe of surveyed facilities. There appears to be a positive association between the number of different surgical procedures provided by a facility and the functionality of that facility (Figure 2)." Comma rather than a full stop between the two sentences might read better.

Response: Changed full stop to comma.

6. Table 6: USD conversion might make this easier for the international reader, especially given changing currency conversion rates. Perhaps both INR and USD figures can be included in sub-columns, using a standard method for conversion (mid point for the study period, or mean conversion rate for the months that the data collection was conducted). Also, can the table be divided by subheadings into overall, government and private sections? That would make it easier for the reader to see at a glance.

Response: Please refer to the response on page 9 in relation to USD conversions.

7. Is there any way to estimate the number of surgeons/surgeries from the data collected in this study or from literature from this area? I would assume probably not given the study design, but since it is part of a bigger study, I thought it might be worth asking if this data is available or possible to extrapolate.

Response: Thank you for this observation, which we will bear in mind. We think it could be possible to report total surgeons and surgeries (general not disaggregated) from the survey/DHS data from the Ensor et al. 2020 paper. However, we are of the view that this information may not be entirely necessary given the aim and scope of this manuscript.

**Reviewer #7: Thank you for the opportunity to review this paper.**

I only have one comment:

this paper does not include evidence of legitimate ethical approval in India. The authors stated that the approval was obtained from Sigma Research and Consulting. I have taken the liberty to look at their website and this is a private consulting company, and no information about whether they are a recognised body to grant ethical approvals is included. This disqualifies this paper from the possibility of being published in a reputable journal because in-country approval is a prerequisite to collecting data in India. Sigma Research and Consulting is not listed as an accredited REC in India.

Before this paper can be reviewed please provide evidence that the study has been carried out in accordance with good practice in research.

Response: Thank you for your response and for highlighting this. We appreciate your concerns and are happy to provide further information on this.

At the time of this study, there were no identifiable Institutional Review Boards operating in Arunachal Pradesh, Nagaland, or Manipur states; with IRBs in Assam focusing primarily on bio-medical research, with remits limited to Assam state. Given the anthropological aspects of the research, as well as its regional focus, advice was sought from the Tomo Riba Institute of Health and Medical Sciences (TRIHMS) in Arunachal Pradesh, as well as the Nagaland Health Project (NHP), in the selection of an IRB, and Sigma IRB came highly recommended. Sigma IRB while located in New Delhi, covers the entire country for IRB-related work. Sigma is registered with the NIH in the USA: please see: [Office for Human Research Protections Database (nih.gov)](https://ohrp.cit.nih.gov/search/search.aspx) (please click to access hyperlink), and focuses on social and behavioural studies. When Sigma IRB was started, ICMR registration was not yet open.

Compliance with European General Data Protection Regulation (GDPR) regulations was ensured. Data was thoroughly anonymised, analysed and encrypted before transmission overseas.

**Reviewer #8:**

Thank you for the opportunity to review this very relevant context specific paper on the surgical capacity and barriers to accessing surgical care in North east India. This paper shows the strength of collaboration across two countries to improve research on this very important topic. The paper is done as a mixed methods study. The Quantitative aspect which was conducted as interviews helps to get the practical unmet needs of the community and the surgical workforce and this helps to identify the solutions to solve them as well.

Introduction: presented well

Methodology: For the facility survey was a standardized tool used? If the tool was not a standardized one, how was it validated? The 6 domains have been explained well. In one of the domains which is regarding the availability of basic surgical equipment 44 items have been ,mentioned. It would be useful to have it listed as an appendix or in the methodology.

Response: Yes, on page 4, we have inserted an additional statement to clarify that along with data from surgical records and log books, a standardised questionnaire based on the WHO Surgical Assessment Tool (SAT), which is mostly composed of closed questions with yes/no, categorical and numeric answers.

Results: Presented adequately. In Table 3, the list of surgical procedures done at these centers have been listed. It would be useful to classify the procedures as essential, non essential and emergency as per the Disease Control Priority list (DCP 3). That would enable the reader to understand the nature of the procedures that the center is equipped to handle.

Discussion: presented well. There is a mention of how the paper shows the readiness for adoption of new surgical innovations. But there is no variable highlighted in the methodology or the results to substantiate the same. Was a related question asked in the interviews that were conducted? If that was done, it will be useful to mention regarding the related question asked and the response got in the results.

Response: Thank you, we have inserted information in both the methodology (pg 4) and the results section ( pg 8-9) to substantiate this.

Limitations: Presented well. The sampling could have been done better to have a near equal representation from the public and the private sector to eliminate the bias. This can be mentioned in the limitation as the majority of the responses were from the Private sector.

Response: Thank you, noted and a couple of sentences added in on pg 13 to clarify the limitation and mention that the majority of the responses were from the private sector.

Conclusion: The recommendations and the solution presented needs to be separated under another heading. As they discuss points that have not been studied in this particular paper.

Response: Noted and the conclusion and recommendations have been separated under another heading.

References: Adequate

6. PLOS authors have the option to publish the peer review history of their article (what does this mean?). If published, this will include your full peer review and any attached files.

If you choose “no”, your identity will remain anonymous but your review may still be made public.

Do you want your identity to be public for this peer review? For information about this choice, including consent withdrawal, please see our Privacy Policy.

Reviewer #1: Yes: Professor Dhananjaya Sharma

Reviewer #2: Yes: Vijay Anand Ismavel

Reviewer #3: No

Reviewer #4: No

Reviewer #5: Yes: Siddhesh Zadey

Reviewer #6: Yes: Lubba Samad

Reviewer #7: No

Reviewer #8: No

[NOTE: If reviewer comments were submitted as an attachment file, they will be attached to this email and accessible via the submission site. Please log into your account, locate the manuscript record, and check for the action link "View Attachments". If this link does not appear, there are no attachment files.]

While revising your submission, please upload your figure files to the Preflight Analysis and Conversion Engine (PACE) digital diagnostic tool, https://pacev2.apexcovantage.com/. PACE helps ensure that figures meet PLOS requirements. To use PACE, you must first register as a user. Registration is free. Then, login and navigate to the UPLOAD tab, where you will find detailed instructions on how to use the tool. If you encounter any issues or have any questions when using PACE, please email PLOS at figures@plos.org. Please note that Supporting Information files do not need this step.

In compliance with data protection regulations, you may request that we remove your personal registration details at any time. (Remove my information/details). Please contact the publication office if you have any questions.
