# Supplementary material for: How ready is the health care system in Northeast India for surgical delivery? a mixed-methods study on surgical capacity and need
Source: PLoS One. 2024 Jun 26;19(6):e0287941. doi: 10.1371/journal.pone.0287941 (PMC11206862; doi:10.1371/journal.pone.0287941)
Supplement: Attachment — Submitted filename: Response to Reviewers-16 Dec 2023.docx [file pone.0287941.s004.docx]

# Response to Reviewers-16 December 2023

Subject: PLOS ONE Decision: Revision required [PONE-D-23-17522R1]

How ready is the health care system in North East India for surgical delivery? A mixed methods study on surgical capacity and need.

Dear Dr. Bains,

Many thanks to you and the reviewers for the careful consideration of our revised manuscript, and for your valuable comments.

As requested, we have appended the following documents,

- Response to reviewers (below)
- A marked-up copy of the manuscript that highlights changes made to the original version, labeled 'Revised Manuscript with Track Changes'.
- An unmarked version of the revised paper without tracked changes, labeled 'Manuscript'.

We believe we have now addressed the reviewers’ comments, and very much look forward to a positive review of this revised version.

Kind regards,

Amrit Virk (on behalf of all authors)

**Additional Editor Comments:**

The authors have done significant improvements in the manuscript as per the reviewer's suggestions. The reviewers have accepted majority of the corrections. Few concerns remaining are listed.

1. IRB approval : Authors are required to provide proper approval documents and clarifications in this regard.
2. Do provide the unique registration number for the mentioned IRB.

Response: We have attached the (i) Sigma approval letter and invoice, both listing the IRB Number 10077/IRB/D/18-19, as well as (ii) a Word document with research where SIGMA approval was used.

SIGMA are registered as an IRB with the Office for Human Research Protections (OHRP) which is a US database of recognised IRBs – see https://ohrp.cit.nih.gov/search/irbsearch.aspx?styp=bsc IORG Number IORG0008260. Importantly, the OHRP database mentioned above is the standard used by the University of Leeds (UK) ethics committee as well, in order to ensure compliance and determine whether in-country ethics boards are recognised.

Our team recognizes the criticality of adhering to ethical standards and addressing IRB approval concerns. And we respect the attention this issue has received from reviewers.

We partnered with the Highland Institute, known for its independent research in indigenous communities and fluency in 16 local languages. Their previous collaboration with the Nagaland Health Project (Ministry of Health) involved both extensive surveys and in-depth ethnographic research, demonstrating significant breadth and depth, but also anthropological expertise. SIGMA IRB's national scope and experience in various Indian states made them a fitting choice for our study's needs. Our study, focusing on sociocultural and behavioural aspects, diverged from the typical biomedical focus of ICMR guidelines, which follow a framework centred on clinical trials, biological samples, and genetic data. Our international collaboration centred on sociocultural research, did not necessitate HMSC approval. All raw data was securely housed at the Highland Institute and shared internationally only after thorough transcription, translation from as many as seven languages, analysis, and anonymization, upholding strict ethical standards. Our method, reflective of medical anthropology, adheres to research ethics, sharing only processed data internationally, as is common in global research collaborations.

# Reviewer's Responses to Questions

Comments to the Author

6. Review Comments to the Author

Please use the space provided to explain your answers to the questions above. You may also include additional comments for the author, including concerns about dual publication, research ethics, or publication ethics. (Please upload your review as an attachment if it exceeds 20,000 characters)

**Reviewer #1:** Authors have addressed one of my comments by including two local authors but the crucial matter of non-availability of "local" ethical committee clearance remains and the final call on this very important issue has to be taken by the Journal

Response: Thank you. Our team recognizes the criticality of adhering to ethical standards and addressing IRB approval concerns. And we respect the attention this issue has received from reviewers.

We have attached the (i) Sigma approval letter and invoice, both listing the IRB Number 10077/IRB/D/18-19, as well as (ii) a Word document with research where SIGMA approval was used.

SIGMA are registered as an IRB with the Office for Human Research Protections (OHRP) which is a US database of recognised IRBs – see https://ohrp.cit.nih.gov/search/irbsearch.aspx?styp=bsc IORG Number IORG0008260. Importantly, the OHRP database mentioned above is the standard used by the University of Leeds (UK) ethics committee as well, in order to ensure compliance and determine whether in-country ethics boards are recognised.

We partnered with the Highland Institute, known for its independent research in indigenous communities and fluency in 16 local languages. Their previous collaboration with the Nagaland Health Project (Ministry of Health) involved both extensive surveys and in-depth ethnographic research, demonstrating significant breadth and depth, but also anthropological expertise. SIGMA IRB's national scope and experience in various Indian states made them a fitting choice for our study's needs. Our study, focusing on sociocultural and behavioural aspects, diverged from the typical biomedical focus of ICMR guidelines, which follow a framework centred on clinical trials, biological samples, and genetic data. Our international collaboration centred on sociocultural research, did not necessitate HMSC approval. All raw data was securely housed at the Highland Institute and shared internationally only after thorough transcription, translation from as many as seven languages, analysis, and anonymization, upholding strict ethical standards. Our method, reflective of medical anthropology, adheres to research ethics, sharing only processed data internationally, as is common in global research collaborations.

Reviewer #2: 1. The sample size is too small to reflect the reality of surgical services in northeast India. This has not changed in the revised manuscript. It is suggested that more centers with more interviews with surgeons and patients be conducted to make this study more representative. Interviewing several surgeons from the same center is unlikely to add information. Interviews can be done through phone calls or videoconferencing without physically visiting the centers.

Response: Thank you for this suggestion, which we will consider in case of future iterations of this work. The purpose of qualitative interviews in general, and for this study in particular, was to explore the issue of surgical services in greater depth, and complement the information emanating from the quantitative component. The aim is not to generalise or claim to be representative; instead, we aimed to illuminate and provide deeper insights into the challenges and opportunities for improved surgical provisioning.

2. There are several references to gasless laparoscopy. How many surgeons interviewed perform gasless laparoscopy? What is their experience (number of surgeries performed and outcome)? In most settings, if a surgical team is able to do laparoscopic surgery, getting carbon dioxide gas is not a problem - it is cheap and easy to obtain in most places where oxygen is obtained. Pneumoperitoneum increases working space, visibility and reduces risk.

Response: Thank you, this clinical information is duly noted. A few surgeons in the sample had received training in the technique of gasless laparoscopy, but since this is not a mainstream service, we explored amenability to this technique on the part of surgeons, but were not aiming to study the association between the number of surgeries performed and the outcome. Notably, there was no specific comparison made regarding the number of surgeries conducted using either Gasless procedures and traditional laparoscopic procedures.

3. There is a mention of dual-position surgeons who work in both government and private facilities. The absence of surgeons full-time in government facilities and referral of patients to private facilities (where often the same surgeons operate) affects patient access to affordable surgical care. Suggested solutions to the issues raised have not been made.

Response: Thank you for this very pertinent point. Given this was not a widely observed pattern in the sample of interviewed providers, we deliberately focused on more prominent and evident discussion points related to the main aim of understanding barriers and opportunities for improving surgical care.

4. Justified risk-taking should be elaborated. When is it acceptable to take risks in remote settings? If an anesthesiologist is not available or adequate blood is not available, is it justified to take risks or refer to a higher center many hours by road at additional expense?

Response: Thank you. As per the suggestion from the reviewer in the earlier iteration, we referenced to the related study that discussed that adaptative approach, (page 30, lines 469-475), providing a reference point for readers interested in deeper engagement with the issue.

5. Readiness for surgical services in northeast India is affected by the poor availability of supporting consultants (compared to other parts of India) - differences between northeast India and the rest of the country has not been described. Government legislation that requires a pathologist to register a blood bank and a radiologist to get PNDT certification (to use ultrasonography) should be relaxed/exempted in settings where such consultants are not available. Nurse anesthetists work in many resource-poor settings across the world - this could be considered.

Response: Thank you for this. We have inserted a few sentences conveying this on page 31, lines 489-493.

6. Northeast India has many locations where road travel is very difficult or not possible during the monsoon. Patients are evacuated at reasonable cost by helicopter if they can be brought to the nearest helipad (especially in states like Arunachal Pradesh). However, this may not be possible when the weather is bad or when there is an unscheduled emergency. This factor affects readiness to access surgical services and should be described.

Response: Thank you. We have inserted this information on page 4, lines 66-70.

7. Surgeons (and other consultants) do not want to work in remote areas because of perceived poor personal and professional life. How many allotted posts for surgeons remain vacant in the region? When a surgeon joins a remote location, how many days is he present on duty? (it is likely that many surgeons do not stay in their remote locations).

Response: Across the survey there was an average of 10% doctor (not surgeon) vacancies at the time of the survey. The main reason for doctors to leave is to acquire more training. Information on hours a doctor is present is not available.

8. Time (and life) is lost in transit. Readiness can be assessed by how often a patient who should have been managed effectively is referred 'to a higher center'. What are the reasons for referral?

Response: Thank you for this important observation. The section on ‘Referrals’ (page 22-25, lines 331-382) and under the ‘Discussion’ (Pages 31-32, lines 507-523) discuss the issue of referrals and influencing factors.

9. Northeast India has large cities with large medical colleges and private hospitals. To generate data that represents the entire northeast, these should also be included in the study.

Response: Thank you. Our sample was purposive since we focused on those organisations from which staff that joined the surgical training programme came. Hence, thereby, we aimed to provide insights into the challenges and opportunities for improving care.

10. For the greatest impact on surgical care delivery, what are the recommendations for prudent investment in facility development, human resources and financial support?

Response: As indicated in the manuscript discussion, prudent investment in minimally invasive surgery including equipment and training as well as blood banking seem to be key.

Reviewer #5: Authors have satisfactorily reworked the manuscript. I just have one comment - in the map added to the revised version, J&K and Ladakh seemed to have cut-off. I am unsure if it is an issue with the PDF copy that was generated or something else. But it needs to corrected.

Response: Thank you for noting this. We have updated the map on page 6.

Reviewer #8: Thank you for the detailed responses to the comments and incorporating the suggested comments. It reads better now.

For the abstract results section, the quantitative results could be incorporated to support the statements mentioned. That would give a better perspective to the reader.

Kindly run the document through a spell check, there are places in the Introduction where 2015 is depicted as 2105,etc.

Response: We have updated the abstract on page 1-2. We have proof-read and made the necessary change on page 3. Thank you for picking this up, and bringing it to our attention.
